# Caseous Calcification of the Mitral Annulus: A Rare Cause of Intracardiac Mass

**DOI:** 10.1155/2012/596962

**Published:** 2012-12-02

**Authors:** Anxo Martinez-de-Alegria, Jose Rubio-Alvarez, Sandra Baleato-Gonzalez

**Affiliations:** ^1^Department of Radiology, University Hospital of Santiago de Compostela, A Coruña, Spain; ^2^Department of Cardiac Surgery, University Hospital of Santiago de Compostela, A Coruña, Spain

## Abstract

Caseous calcification of the mitral annulus is a rare form of periannular calcification with a mass-like appearance, that has to be in the differential of the cardiologist and radiologist. It classically looks like a round or semilunar hyperdense mass with an even denser peripheral rim, located in the posterior mitral annulus and having in general no clinical significance.

## 1. Case Presentation

**Figure 1:**
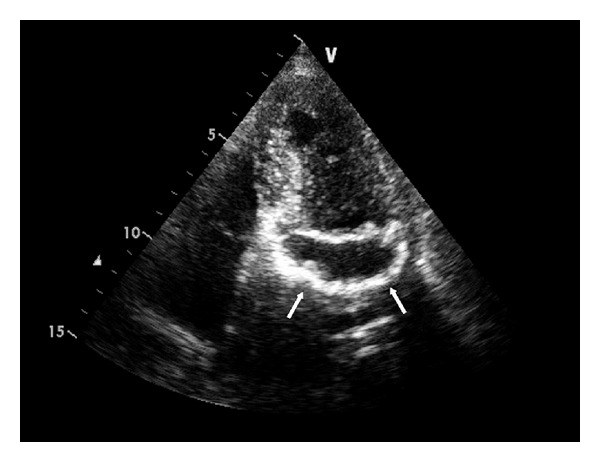
Echocardiographic apical four-chamber view showing a large oval echolucent mass with echodense rim in the mitral annular region.

**Figure 2:**
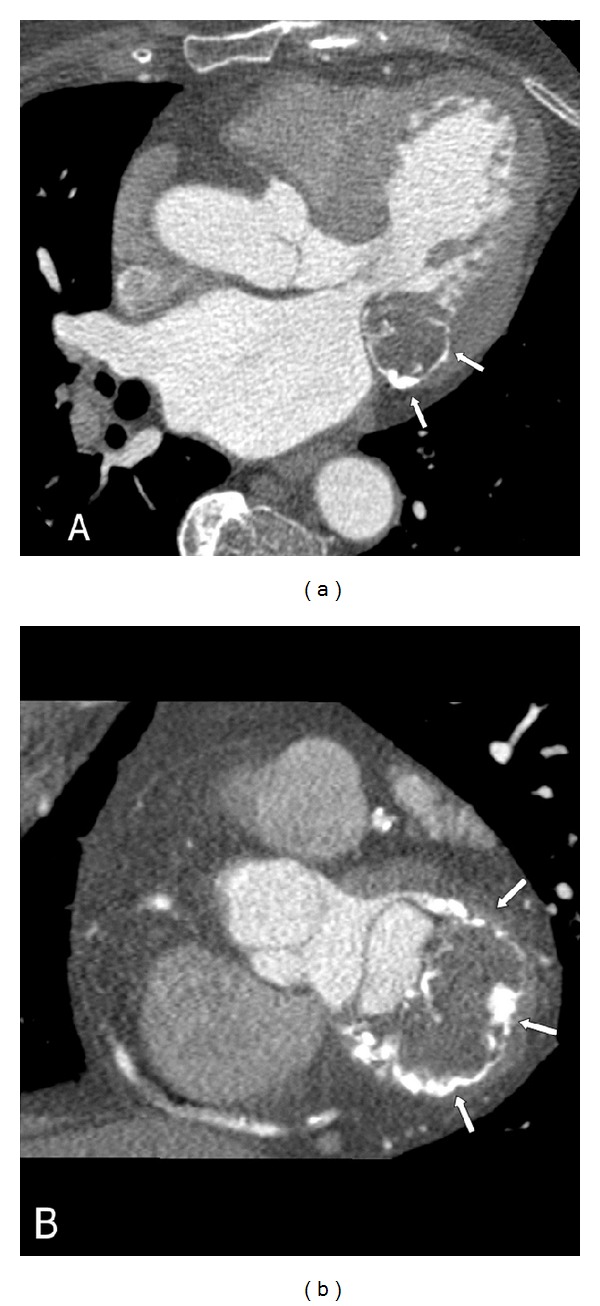
Cardiac CT, long axis (a) and short axis (b) views, showing a crescent-shaped hypodense mass with peripheral linear calcification that produces a shifting of posterior leaflet of mitral valve. No alterations of valvular function were found.

A 76-year-old woman with atrial fibrillation and hyperlipidemia underwent a routine echocardiography. She presented a round cistic lesion with echodense rim in the mitral valve annulus (Figure 1), not associated to valvular dysfunction or related symptoms. A ECG-gated cardiac CT was performed (Figure 2) that showed a semilunar mass with hypodense center and a calcified peripheral rim, locally thickening the inferior and posterolateral basal left ventricular wall. The central content had homogeneous fluid density and no significant contrast enhancement.

After ruling out clinically abscessified mitral calcification, the imaging findings were considered highly suggestive of caseous calcification of the mitral annulus. Surgery was not needed due to the absence of coexistent mitral valve lesions and the patient was followed up conservatively, showing no echocardiographic changes after one year.

## 2. Discussion

Caseous calcification of the mitral annulus, also called liquefaction necrosis, is a chronic degenerative process of the mitral valve fibrous ring, primarily involving the posterior annulus. It is a common disorder in the elderly, mainly in women, observed at autopsy in 8% of the population but rarely seen in imaging [1]. As an expression of atherosclerosis, it has identical risk factors as cardiovascular disease [2].

The precise mechanisms involved in the caseous calcification of mitral annulus are not known, but it is at present considered a dynamic entity. In different published reports of patients followup, some cases of caseous calcification disappeared spontaneously, replaced by simple annular calcification, whereas other patients with common annular calcifications developed central echolucent zones compatible with caseous calcification [3, 4].

The typical echocardiographic appearance of caseous calcification of the mitral annulus is a large round echogenic mass with a central echolucent area resembling liquefaction, located at the posterior side of the mitral valve annulus. It looks very different from the C or U morphology of the dense echo band in common mitral annulus calcification. Caseous calcification is less reflective than common calcification of mitral annulus and is usually described as with no acoustic shadowing artifacts behind it [5].

On multislice CT, this entity appears as a round or semilunar mass with an hypo- or hyperdense center and a calcified peripheral rim. Heterogeneity of the content is confirmed by the varying densities that can be seen, ranging from negative Hounsfield units suggesting fatty degeneration to dense material [6].

When surgery is performed, a calcific shell is usually detected surrounding a central area filled with a toothpaste-like material composed of calcium, fatty acids, and cholesterol. Depending on which of those predominate, the density of the mass content may vary in CT from “milky” to fluid or even fat. Histological analysis reveals a pasty acellular substance that is culture negative [7].

The differential diagnosis of a semilunar peripherally calcified structure in the mitral annulus includes mainly infected or abscessified mitral calcification, calcified tumor, and hydatid cyst. The distinction between caseous calcification and mitral annular abscess should be based on the different clinical presentation, and the lack of a large amount of calcification in the latter. Regarding tumors, intramural cardiac myxoma is rare and primary cardiac osteosarcoma usually originates in the left atrium. Cardiac hydatid cyst is a rare entity.

There is no consensus on the optimal treatment of caseous mitral annular calcification. Although some data in the literature indicate that it is associated with an increased risk of stroke, several patients followed after conservative treatment did not suffer stroke [5]. Surgery, therefore, should be probably reserved for coexistent mitral valve lesions.

Unlike common mitral annular calcification, asymmetrical tumor-like caseous calcification has been described as a rare condition. But with the increasing use of cardiac CT, this incidental lesion may be more commonly encountered in clinical practice and cardiac imagers should be familiar with it in order to avoid misdiagnosis.
